# Dexamethasone-induced impairment of post-injury skeletal muscle regeneration

**DOI:** 10.1186/s12917-019-1804-1

**Published:** 2019-02-11

**Authors:** Iwona Otrocka-Domagała, Katarzyna Paździor-Czapula, Michał Gesek

**Affiliations:** Department of Pathological Anatomy, Faculty of Veterinary Medicine, University of Warmia and Mazury, Oczapowskiego Street 13, 10-719 Olsztyn, Poland

**Keywords:** Dexamethasone, Glucocorticoid, Skeletal muscle regeneration, Regenerative capacity impairment, Porcine model

## Abstract

**Background:**

Due to the routine use of dexamethasone (DEX) in veterinary and human medicine and its negative impact on the rate of wound healing and skeletal muscle condition, we decided to investigate the effect of DEX on the inflammatory and repair phases of skeletal muscle regeneration. In this study, a porcine skeletal muscle injury model was used. The animals were divided into non-treated and DEX-treated (0.2 mg/kg/day) groups. On the 15th day of DEX administration, bupivacaine hydrochloride-induced muscle injury was performed, and the animals were sacrificed in subsequent days. Regeneration was assessed by histopathology and immunohistochemistry. In the inflammatory phase, the presence and degree of extravasation, necrosis and inflammation were evaluated, while in the repair phase, the numbers of muscle precursor cells (MPCs), myotubes and young myofibres were estimated.

**Results:**

In the inflammatory phase, DEX increased the severity and prolonged extravasation, prolonged necrosis and inflammation at the site of the muscle injury. In the repair phase, DEX delayed and prolonged MPC presence, impaired and prolonged myotube formation, and delayed young myofibre formation. Furthermore, DEX markedly affected the kinetics of the parameters of the inflammatory phase of the skeletal muscle regeneration more than that of the repair phase.

**Conclusions:**

DEX impairment of the inflammatory and repair phases of the skeletal muscle regeneration was proven for the first time. The drug appears to affect the inflammatory phase more than the repair phase of regeneration. In light of our results, the possibility of reduction of the regenerative capacity of skeletal muscles should be considered during DEX therapy, and its use should be based on risk–benefit assessment.

## Background

Dexamethasone (DEX) belongs to the steroidal drugs (also known as glucocorticosteroids, glucocorticoids or corticosteroids), and its main actions are immunosuppressive, anti-inflammatory and anti-allergic effects [1]. The anti-inflammatory properties of DEX consist of inhibiting the expression of pro-inflammatory cytokines, chemokines and growth factors and enhancing endothelial NO production, resulting in decreases in the recruitment and migration of neutrophils and macrophages to inflammatory sites [2, 3]. Glucocorticoids (GCs), due to their anti-inflammatory properties, are well-known factors regulating multiple aspects of endothelial physiology, including the expression of adhesion molecules, production of pro-inflammatory cytokines and chemokines, and preservation of the endothelial barrier integrity [3, 4]. However, Iuchi et al. [5] showed that GCs may elicit vascular endothelial dysfunction by inducing the overproduction of reactive oxygen species (ROS) by endothelial cells. Therefore, because of the alarming results of other studies, recent investigations have raised concerns regarding the potential negative effects of long- and short-term DEX therapy on the cardiovascular system, wound healing and skeletal muscle condition [6, 7]. Studies on humans and laboratory animals have shown that the impairment of wound healing during DEX administration probably occurs at the early stage of the process through a reduction in the numbers of macrophages and neutrophils and subsequent delay in cellular debris removal, as well as through down-regulation of interleukins, cytokines, chemokines and growth factors at the injury site [7–9].

In relation to the skeletal muscle, it was observed that DEX increased protein breakdown in young adults, decreased protein synthesis in aged rats, and morphologically and functionally damaged muscle precursor cells (MPCs; satellite cells, myoblasts) [6, 10]. Moreover, GCs can induce apoptosis in skeletal myofibres by activating mitochondrial and FAS-dependent pathways [11]. Furthermore, DEX impairs the proliferation activity and regeneration capacity of myoblasts and enhances their apoptosis [12]. In contrast, some in vitro studies showed that DEX improved myogenesis and membrane repair in dosage-depend manner by enhancing mouse MPC proliferation, differentiation and fusion to myotubes, potentially through the induction of dysferlin, a calcium-binding transmembrane protein that plays a key role in both processes [13, 14]. In general, it should be considered that DEX can undermine MPC recruitment, activity and differentiation, resulting in restraining of the repair phase of post-injury skeletal muscle regeneration [11, 12].

Skeletal muscle fibres have a unique ability to regenerate, which depends on their structure, function and condition. Skeletal muscle regeneration consists of two general phases: the degeneration phase, with an inflammatory response, and the myofibre repair phase, with activation, differentiation and fusion of MPCs and young myofibre formation [15]. The first phase is dependent on the secretion of cytokines, chemokines and growth factors at the site of the muscle injury and is accompanied by infiltration of neutrophils, followed by pro-inflammatory (M1) macrophages and then anti-inflammatory (M2) macrophages. This phase of the regeneration is closely linked to the second phase because inflammatory cells, fibroblasts and extracellular matrix of damaged tissue release chemotactic signals that activate the proliferation and early differentiation of MPCs [16, 17]. Furthermore, MPCs themselves attract inflammatory cells, mainly macrophages, to the site of post-injury regeneration and use them as a support to escape apoptosis, especially during their fusion [18, 19].

Considering the above results, the regeneration of skeletal muscle is a complex process regulated by various mechanisms such as cell-cell and cell-matrix interactions, as well as the secretion of many factors coordinating inflammatory and MPC activity. Unfortunately, these interactions can be impaired by diseases, ageing and potentially myotoxic and non-myotoxic drugs, resulting in lowering of the regenerative capacity of skeletal myofibres. A potential factor that could have such effects is DEX. Based on the available literature, whether this glucocorticosteroid treatment affects the inflammatory and repair phases of skeletal myofibre regeneration – in terms of undesirable consequences – is unclear. Clarifying this issue is very important because DEX is widely used to treat many conditions, especially post-traumatic conditions, in infants, young adults and elderly animals and humans, and it will be beneficial to avoid tissue regeneration disorders during glucocorticosteroid therapy.

In light of the above-mentioned GCs properties, a hypothesis that DEX can impair the course of post-injury skeletal muscles regeneration was assumed. We hypothesized that DEX: (a) can disturb the inflammatory phase by inhibiting of the inflammatory response of the injured tissue and reducing the recruitment of inflammatory cells; (b) may negatively affects the repair phase of the post-injury skeletal muscle regeneration by undermine MPC recruitment, activity and differentiation. In order to verify these hypotheses, the purpose of this study was to determine the effect of DEX treatment on the course of the inflammatory and repair phases of experimentally injured skeletal muscle regeneration. To investigate the mechanisms underlying the potential anti-regenerative effect of DEX the following parameters were analysed: (a) the presence and degree of extravasations, (b) the presence and degree of necrosis, (c) the presence and intensity of inflammation, (d) the mean number of MPCs, (e) the mean number of myotubes, and (f) the mean number of myofibres.

## Results

### DEX increases the severity and prolongs extravasation in BPVC-injured muscles

**Fig. 1:**
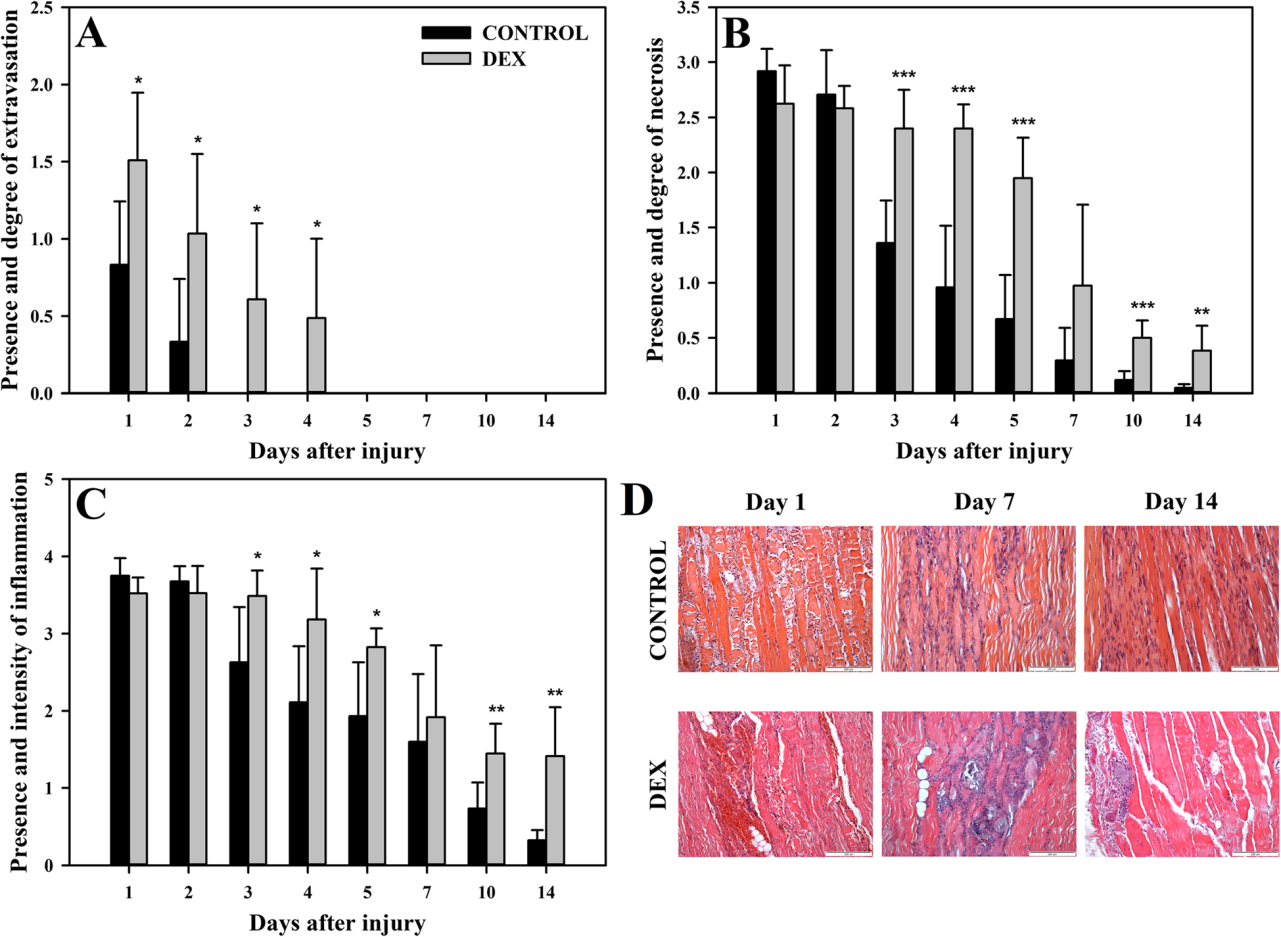
The effect of DEX on the inflammatory phase of post-injury skeletal muscle regeneration. To establish this effect, the presence and degree of extravasation (**a**), the presence and degree of necrosis (**b**), and presence and intensity of inflammation (**c**) were evaluated in the BPVC-injured muscles of non-treated (control) and DEX-treated animals. The results are expressed as the mean score (± SD) from 6 sites of muscle injury derived from 3 animals (2 independent muscle injuries per single animal) per group per day. The *P*-values (^∗^*p* < 0.05, ^∗∗^*p* < 0.01, ^∗∗∗^*p* < 0.001) refer to the significant differences between groups at the same time point. Representative H&E stained sections of post-injury myofibre regeneration sites. Day 1; Control: extensive necrosis, focal extravasation, and moderate inflammation. DEX: extensive necrosis and extravasation and moderate inflammation Day 7; Control: moderately numerous myotubes and numerous young myofibres. DEX: mild inflammation, numerous myotubes, and moderately numerous young myofibres. Day 14; Control: numerous young myofibres. DEX: Focal necrosis, mild inflammation, not numerous myotubes, and moderately numerous young myofibres (**d**)

**Fig. 2:**
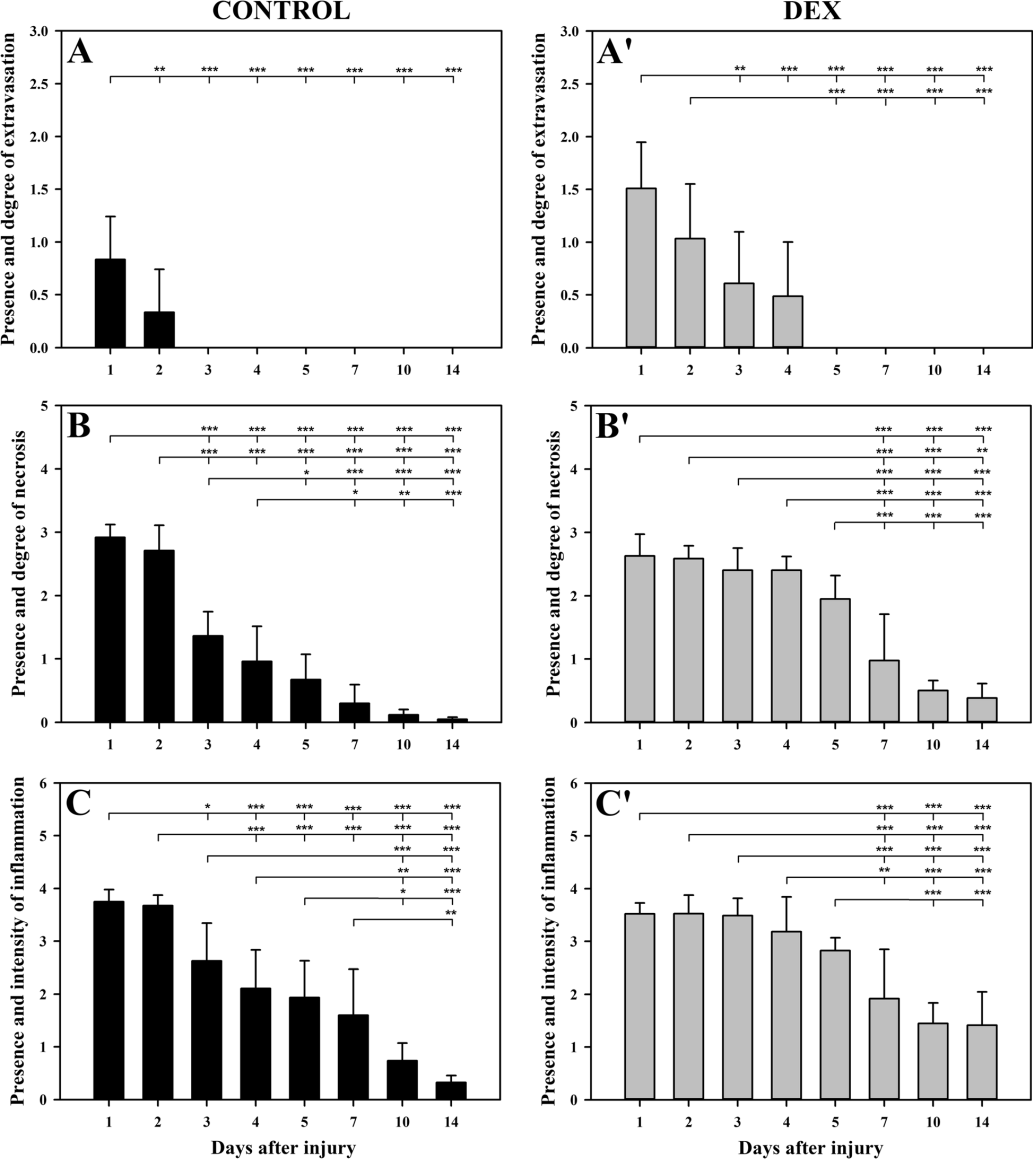
The effect of DEX on the kinetics of the inflammatory phase parameters of post-injury skeletal muscle regeneration. To establish this effect, the presence and degree of extravasation (**a**, a’), the presence and degree of necrosis (**b**, b’), and presence and intensity of inflammation (**c**, c’) were compared between particular time points in the BPVC-injured muscles of non-treated (control) or DEX-treated animals. The results are expressed as the mean score (± SD) from 6 sites of muscle injury derived from 3 animals (2 independent muscle injuries per single animal) per day in the control (**a**, **b** and **c**) and DEX (a’, b’ and c’) groups. The *p*-values (^∗^*p* < 0.05, ^∗∗^*p* < 0.01, ^∗∗^*p* < 0.001) refer to the significant differences among all time points in the control group or DEX group

Treatment with DEX resulted in significant increase (*p* < 0.05) in the distribution of extravasation on day 1 compared with the control group (Fig. 1a, d). Similarly, on days 2, 3 and 4, this parameter was significantly higher (*p* < 0.05, *p* < 0.05, *p* < 0.05, respectively; Fig. 1a) in the DEX-treated animals than in the control group. These results indicated that DEX increased the severity and extended the period of extravasation in BPVC-injured muscles. Comparative analysis of the kinetics of this parameter in DEX-treated and non-treated animals at all analysed time points also confirmed this conclusion (Fig. 2a, a’). In the DEX group, the distribution of extravasations did not significantly decrease until day 4, while in the control group, this parameter was already markedly reduced on day 2 (*p* < 0.01), and at the third time point, extravasations were not observed in the control animals (Fig. 2a, a’). Therefore, it was concluded that DEX extended the period of extravasations in a significant manner.

### DEX prolongs necrosis in BPVC-injured muscles

DEX treatment did not affect the degree of necrosis on days 1 and 2; nevertheless, on days 3, 4, 5, 10 and 14, the mean extent of necrosis was considerably higher in DEX-treated animals than in the control group (day 3: *p* < 0.001; day 4: *p* < 0.001; day 5: *p* < 0.001; day 10: *p* < 0.001; day 14: *p* < 0.01; Fig. 1b, d). These results indicated that DEX prolonged necrosis in BPVC-injured muscles. Comparative analysis of the kinetics of necrosis in both groups also supported this conclusion (Fig. 2b, b’). In the DEX group, extensive necrosis was maintained up to day 5 after muscle injury, and its significant reduction (*p* < 0.001; Fig. 2a’) occurred only on day 7 (Fig. 1d, Fig. 2b’); in contrast, in the control group, this parameter already considerably decreased (*p* < 0.001; Fig. 2b) on day 3, and this decline continued until the end of the experiment. These results demonstrated that DEX prolonged necrosis in BPVC-injured muscles.

### DEX prolongs inflammation in BPVC-injured muscles

On days 1 and 2, DEX did not affect the intensity of inflammation compared with that in the non-treated animals (Fig. 1c, d). However, on days 3, 4, 5, 10 and 14, the mean number of inflammatory cells was considerably higher after DEX treatment than that for the control values (day 3: *p* < 0.05; day 4: *p* < 0.05; day 5: *p* < 0.05; day 10: *p* < 0.01; day 14: *p* < 0.01; Fig. 1c). These results indicated that DEX treatment prolonged inflammation in BPVC-injured muscles. Comparative analysis of the kinetics of the mean number of inflammatory cells in the DEX and control groups also confirmed this conclusion (Fig. 2c, c’). DEX maintained inflammation at the same level until day 5 (Fig. 2c), while in the control group, the intensity of inflammation decreased gradually from day 3 to the end of the experiment (Fig. 2c). In the DEX group, a decrease in the severity of inflammation was only observed from day 7, and the severity remained unchanged at the next time points (Fig. 1d, Fig. 2‘). In conclusion, DEX delayed resolution of the inflammation in BPVC-injured muscles. In both groups on day 1, neutrophils were mostly observed in BPVC-injured muscles, while on day 2, macrophages predominated, with a lower share of neutrophils. On day 3, in both groups, macrophages predominated in injured muscles, and neutrophils and lymphocytes were occasionally observed. From day 4 to the last analysed time point in both groups, macrophages predominated in injured muscles with slight participation of lymphocytes and occasionally observed eosinophils.

### DEX delays and prolongs MPC presence in BPVC-injured muscles

**Fig. 3:**
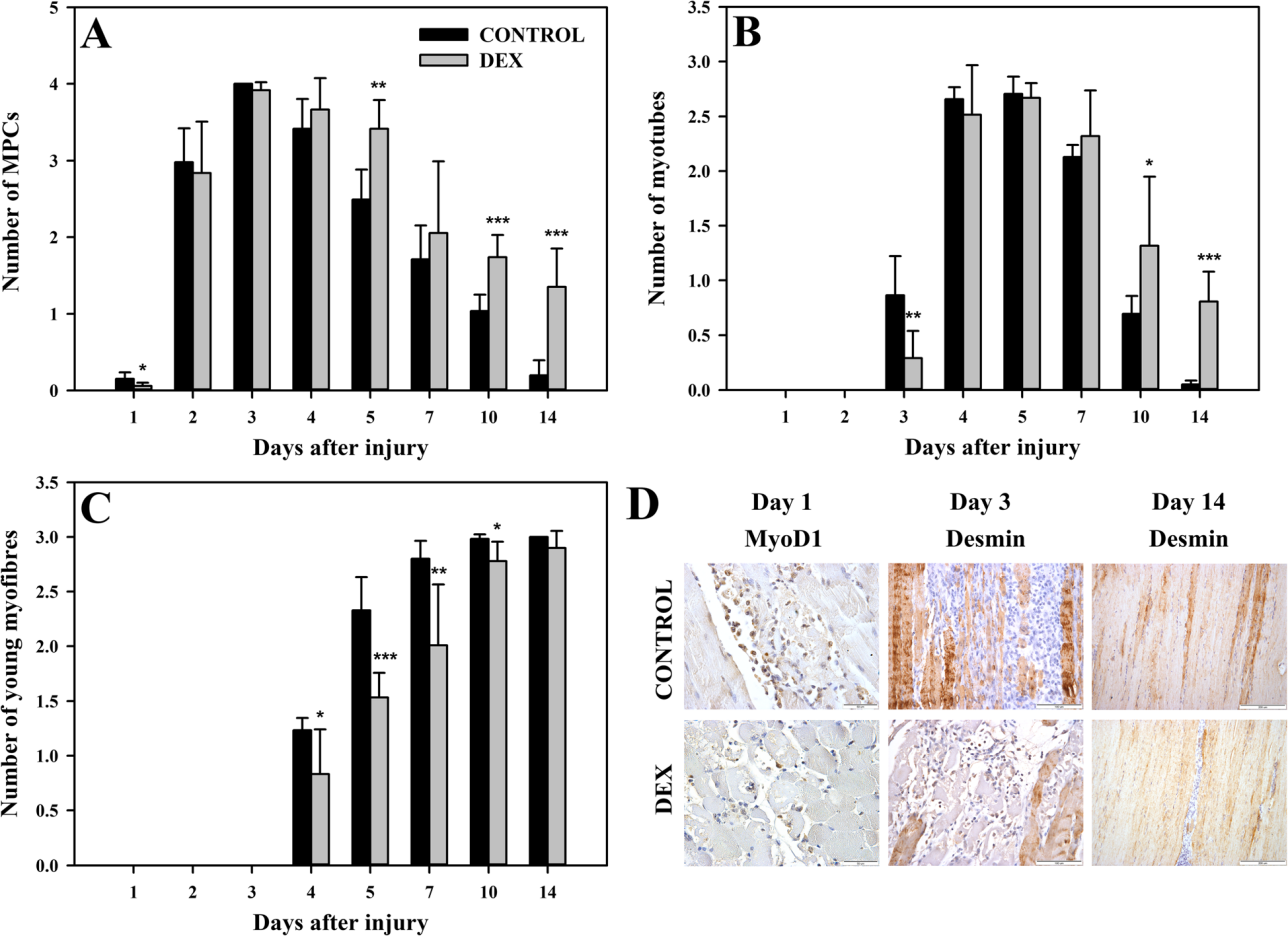
The effect DEX on the repair phase of post-injury skeletal muscle regeneration. To establish this effect, the mean numbers of muscle precursor cells (MPCs) (**a**), myotubes (**b**) and young myofibres (**c**) were evaluated in BPVC-injured muscles of non-treated (control) and DEX-treated animals. The results represent the mean score (± SD) from 6 sites of muscle injury derived from 3 animals (2 independent muscle injuries per single animal) per group per day. The *p*-values (^∗^*p* < 0.05, ^∗∗^ *p* < 0.01, ^∗∗∗^*p* < 0.001) refer to the significant differences between the groups at the same time point. Representative immunohistochemistry of post-injury myofibre regeneration sites. Day 1; Control: MyoD1-positive, not numerous MPCs. DEX: MyoD1-positive, single MPCs. Day 3; Control: desmin-positive, not numerous myotubes and myofibres. DEX: desmin-positive, numerous MPCs and myofibres, and the lack of positive myotubes. Day 14; Control: desmin-positive, numerous young myofibres. DEX: desmin positive, not numerous myotubes and numerous young myofibres. All sections were counterstained with haematoxylin (**d**)

**Fig. 4:**
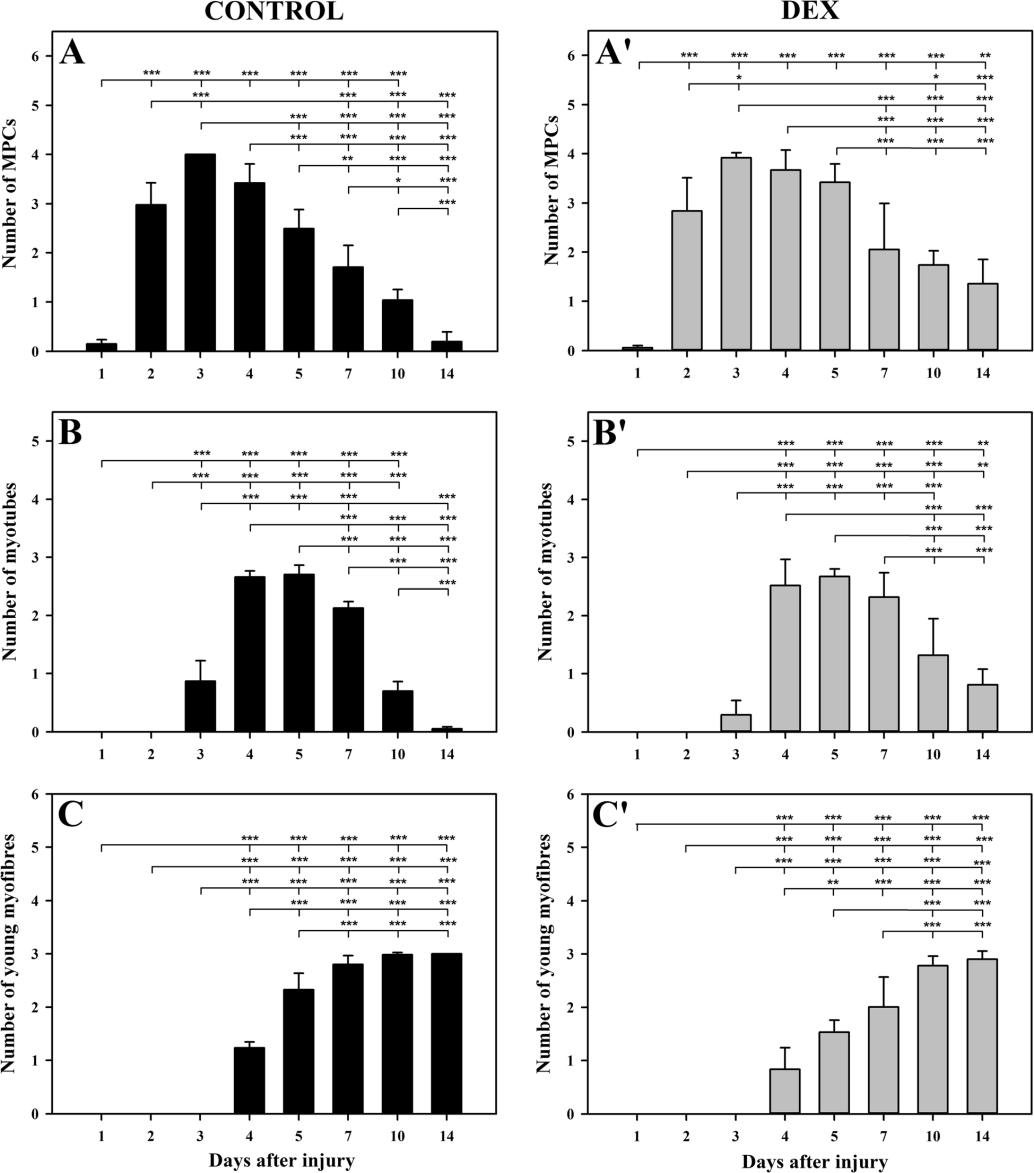
The effect of DEX on the kinetics of the repair phase parameters of post-injury skeletal muscle regeneration. To establish this effect, the mean numbers of muscle precursor cells (MPCs) (**a**, a’), myotubes (**b**, b’) and young myofibres (**c**, c’) were compared between particular time points in the BPVC-injured muscles of non-treated (control) or DEX-treated animals. The results are expressed as the mean score (± SD) from 6 sites of muscle injury derived from 3 animals (2 independent muscle injuries per single animal) per day in the control (**a**, **b** and **c**) and DEX (a’, b’ and c’) groups. The *p*-values (^∗^*p* < 0.05, ^∗∗^*p* < 0.01, ^∗∗∗^*p* < 0.001) refer to the significant differences among all time points in the control group or DEX group

The presence of MPCs was evaluated by H&E staining and was confirmed by the expression of MyoD1 and desmin. DEX treatment caused a significant reduction (*p* < 0.05; Fig. 3a) in the mean MPC number compared to that of the control group on day 1 (Fig. 3d). On days 2, 3, and 4, this parameter did not differ considerably between both groups; however, on days 5, 10 and 14, during DEX treatment, the MPC number was considerably higher (*p* < 0.01, *p* < 0.001, *p* < 0.001, respectively; Fig. 3a) in the treatment group than in the control group. This comparative analysis of the mean MPC number between the groups indicated that DEX delayed and prolonged the presence of MPCs in BPVC-injured muscles. This conclusion was also supported by the observation of the kinetics of this parameter in the DEX and control groups (Fig. 4a, a’). In both groups, the kinetics of the mean MPC number was convergent and continued up to day 7 after muscle injury. Nevertheless, in the next time points, the number of MPCs was significantly reduced in the control group, while in DEX group, it remained unchanged (Fig. 4a, a’); this result indicated that DEX treatment prolongs the presence of MPCs during regeneration.

### DEX impairs and prolongs myotube formation in BPVC-injured muscles

The presence of multinucleated myotubes that were small in diameter with slightly basophilic cytoplasm, was confirmed by H&E staining and desmin cytoplasmic immunoexpression. The first myotubes were observed on day 3 in both groups; however, in the DEX group, the number of myotubes was significantly lower than that in the control group (*p* < 0.01; Fig. 3b, d). On days 4, 5 and 7, this parameter did not differ considerably between both groups, while on days 10 and 14, the number of myotubes was significantly higher in the DEX group than in the control group (*p* < 0.05, *p* < 0.001, respectively; Fig. 1d, Fig. 3b, d). These results proved that DEX impaired and prolonged myotube formation in regenerating BPVC-injured muscle. The analysis of the kinetics of the mean myotube number was similar in both groups; however, a significant difference was observed at the end of the experiment (Fig. 4b, b’). In the control group, from day 7, there was a rapid reduction of myotubes, and on day 14, the myotubes were mostly absent (Fig. 4b). In contrast, in the DEX group on days 10 and 14, the myotube number were maintained at a relatively high level (Fig. 4b’). This result indicated that DEX treatment prolongs the presence of myotubes in regenerating skeletal muscles.

### DEX delays young myofibre formation in BPVC-injured muscles

The first young myofibres with initially centrally located nuclei and a basophilic, desmin-positive cytoplasm were observed on day 4 in DEX-treated and non-treated animals; however, their mean number was significantly reduced (*p* < 0.05; Fig. 3c) after DEX treatment compared with the control values. At the following time points, this parameter was also considerably decreased in the DEX group compared to that in the control group (day 5: *p* < 0.001; day 7: *p* < 0.01; day 10: *p* < 0.05; Fig. 1d, Fig. 3c). On day 14, the mean number of young myofibres did not differ significantly between both groups (Fig. 3c, d). These results showed that DEX treatment delays the formation of young myofibres, which is also supported by comparative analysis of the kinetics of this parameter in both groups (Fig. 4c, c‘). The kinetics of the mean number of young myofibres was similar in the DEX and control groups; however, the scale of new myofibres formation was markedly lower in the DEX-treated animals than in the controls (Fig. 4c, c‘). Therefore, it was concluded that DEX impairs young myofibre formation.

## Discussion

This study investigated the influence of the DEX on the course of post-injury skeletal muscle regeneration. We provided evidence that in the inflammatory phase, DEX increased the severity and prolonged extravasation, prolonged necrosis and inflammation at the site of the muscle injury. In the repair phase, DEX delayed and prolonged MPC presence, impaired and prolonged myotube formation, and delayed young myofibre formation. Furthermore, DEX markedly affected the processes occurring in the inflammatory phase of skeletal muscle regeneration to a greater extent than those in the repair phase.

Destabilization of the endothelial cell barrier by DEX, which was manifested by extensive extravasation and its prolonged presence in the BPVC injection site, was one of the surprising results of our study. Dose-dependent DEX impairment of vascular permeability is controversial because the results of various studies on this topic are contradictory, even if the similar DEX doses, in the range of 0.05 mg/kg - 1.0 mg/kg, were used [3, 20, 21]. It was shown that under hypoxic conditions in acute ischaemic stroke or acute tissue injury, DEX is ineffective or even harmful to the endothelial cell barrier function [3]. This effect is related to the reduction of junctional protein expression, transendothelial electrical resistance (TER) and GS receptors in the ischaemic environment [3]. Similar conditions occur in the BPVC-injured muscle, where hypoxia is the consequence of short-term vasoconstriction, followed by vasodilation with increased blood vessel permeability, which occurs after anaesthetic injection [22]. Based on the above results and taking into account the fact that in our study extravasation was more extensive after DEX treatment than in the control group, it should be assumed under conditions of tissue ischaemia, DEX unfavourably affects the endothelial barrier integrity in skeletal muscles.

In our study, DEX did not affect the severity of necrosis during the first two days of the regeneration. Therefore, it can be concluded that DEX does not prevent muscle damage nor does it exhibit a myotoxic action. This finding is in line with those of previous studies in both laboratory animals and humans [2, 23]. Intriguingly, from day 3 of regeneration to the end of our experiment, myonecrosis was more extensive in the DEX-treated animals than in the non-treated animals; furthermore, it was accompanied by a high number of inflammatory cells, which was also observed from this time point in the DEX group. Surprisingly, during the first two days (the time of neutrophil activity), DEX did not affect the intensity of inflammation, which means that it does not exert any anti-inflammatory effect at the beginning of the skeletal muscle regeneration. Moreover, it appears that DEX induced enhancement of the inflammation in the later inflammatory phase of the regeneration. This result could be due to the anti-apoptotic effect of DEX on neutrophils, that prolonged presence of these inflammatory cells at the site of muscle injury. The anti-apoptotic effect of GCs on neutrophils has been reported by in vitro studies, but the exact mechanisms of this action are still being discussed [24, 25]. We also hypothesized that prolonged inflammation after DEX treatment could be the result of an increase in the macrophage population caused by DEX. This finding is in agreement with those of Kimura et al. [26] and other studies in which higher macrophage exudation was observed after GCs treatment during tissue recovery [27]. According to the latest research results, macrophage migration inhibitory factor (MIF) is one of the factors responsible for promoting macrophage adhesion and migration into tissue during inflammatory responses. MIF also stimulates its own synthesis and the synthesis of other pro-inflammatory mediators [28]. Intriguingly, MIF has a unique relationship with GCs and acts as a counter-regulator to the anti-inflammatory and immunosuppressive activities of GCs, and in some circumstances, its secretion can be induced by GCs and DEX [28, 29]. However, studies on these observations have never been performed in post-injury regenerating skeletal muscle during DEX administration; therefore, further studies to explain these interdependencies are needed.

Surprisingly, the higher number of inflammatory cells after DEX treatment was not accompanied by faster cleaning up of necrosis compared to that in the control group. The explanation for this phenomenon may be that DEX reduced of the macrophage phagocytic activity [30]. These properties of DEX were observed in in vitro and in vivo studies; however, the mechanisms by which GCs decrease the phagocytic function of macrophages are unknown. The reduction fluidity of macrophage membranes, down-regulation of mRNA expression and activity of metalloproteinases and macrophage apoptosis induction are possible mechanisms considered in the control of macrophage differentiation, migration and function by DEX [30–33]. It was also shown that the localization and microenvironment can modulate the effect of DEX on macrophage activity; however, this phenomenon has been never investigated in post-injury regenerating muscle. An additional reason for the slower cleaning of the necrotic debris after DEX treatment, which was observed in our study, is that the glucocorticoid induces a shift of the macrophage phenotype from phagocytic M1 towards non-phagocytic M2. This property of DEX has been shown in the latest research [34]; however, this phenomenon has never been studied in relation to muscle regeneration, and it is not known how it affects the course of this process. Therefore, all these issues regarding macrophages activity, function and differentiation in post-injury skeletal muscle regeneration under DEX exposure must be clarified.

In our study, DEX also significantly impaired the repair phase of the regeneration. We showed that at the beginning of this phase, the drug negatively affected MPC activity, which in turn causes delayed and decreased myotube and young myofibre formation. Our findings are in line with in vitro studies in which the negative effect of DEX on MPC proliferation and differentiation capacity has been achieved at high concentrations of DEX in the range of 0.01 μM - 100 μM [12, 34, 35]. This action of DEX results from the induction of MPC apoptosis, acceleration of protein degradation, and reduction of proliferative and differentiation activity with a subsequent decrease in myotube fusion [12, 35, 36]. However, in other in vitro studies it has been shown that low concentrations of DEX, in the range of 0.005 μM - 0.1 μM, applied once or at intervals can have a beneficial effect on MPCs proliferation and differentiation [13, 14].

Impairment of the myotube and young fibre formation by DEX, which was observed in our research, is in agreement with in vitro studies with the use of high concentrations of DEX [35, 37, 38]; however, other in vitro experiments showed that DEX pre-treatment or treatment in low concentrations during stimulation of myogenesis improved this process [13, 14].

Paradoxically, in our study, until the end of the experiment, the presence of MPCs was accompanied by an increased number of inflammatory cells. We hypothesize that this phenomenon could be the result of DEX induction of the shift of the macrophages from M1 towards M2 phenotype with subsequent persistent stimulation of MPCs.

The results of our study are in some aspects not in line with results from other research on the effect of GCs on tissue regeneration. The mechanisms underlying the positive versus negative effects of DEX on this process are not clearly understood; however, the effects appear to depend on the dose of DEX and the research model used. Unfortunately, dose-dependent comparative study of DEX properties in in vitro and in vivo models is challenging due to the use of different drug units and different methods of cell exposure to GCs. The DEX dosage used in our experiment was not high and close to the upper range of the maintenance anti-inflammatory dose in the target animal species and humans; however, its administration for fourteen days before and during BPVC-induced muscle regeneration could significantly affect the cells involved in this process by direct or indirect mechanisms. We are aware that the limitation of this research is the use of a single dose of DEX. Therefore, further studies are required to determine the dose- and time-dependent effect of DEX on the course of skeletal muscle regeneration. Moreover, the downside of in vitro studies of such complex processes as skeletal muscle regeneration is the inability to observe the mutual correlations of all cell populations participating in it. Therefore, future in vivo research must focus on filling the gap in the scientific literature regarding the effect of GCs on the inflammatory and recovery phases of post-injury skeletal muscle regeneration in animals and humans.

In this study, the BPVC model of muscle injury was used to evaluate the effect of DEX on the course of the myofibres regeneration process. Intramuscular injection of BPVC causes myonecrosis without damage to MPCs and the basement membrane of muscle fibres, followed by rapid regeneration [39]. Because of these advantages, this model of muscle injury is used in research on causes of regeneration failure under various conditions, such as muscular dystrophies [40, 41] or autoimmune myositis [42], and in studies on the positive or negative influence of various substances [43–45] and physical factors [46] on the course of the muscle regeneration and myogenesis.

## Conclusions

The current study showed, that DEX may impair the post-injury skeletal muscle regeneration. Moreover, the results strongly suggest that the inflammatory phase of the regeneration seems to be more affected than the repair phase during DEX therapy. DEX did not exert any anti-inflammatory effect at the beginning of the inflammatory phase, rather it further enhanced the inflammation status during this phase. Furthermore, DEX seemed to reduce of the macrophage phagocytic activity, resulting in prolonged removal of the necrotic debris. In the repair phase, DEX impaired recruitment and differentiation of MPC, resulting in delayed myotube and young myofibre formation. These results have a multi-faceted implication on both veterinary and human medicine, because DEX is routinely used for treatment of similar conditions in animals and humans, especially in tissue and organ trauma. Bearing in mind all observed DEX-induced effects, the possibility of reduction of the regenerative capacity of skeletal muscle should be considered during glucocorticoid therapy. Therefore, its use in patients with muscle trauma should be based on risk–benefit assessment, and further detailed studies of the dose- and time-dependent side effects of DEX therapy are necessary.

## Methods

### Animals and study design

The experiment was conducted on forty-eight clinically healthy gilts (Large White Polish breed), aged 3 months (at the start of the experiment), that were obtained commercially from a large pig farm (Agro Group Sp. z o.o.).

The gilts were housed in the experimental section of the Faculty of Veterinary Medicine, Warmia and Mazury University in Olsztyn. The animals were kept in ventilated 10-m^2^ pens (24 gilts per pen) on a concrete floor with rubber mat areas and a natural light/dark cycle. The pens were cleaned twice a day, and the animals were fed with commercial grower feed twice a day, with ad libitum access to fresh water. During the 10 days of adaptation before the initiation of the experiment, the gilts were kept at 25 °C, and during the experiment, they were kept at 23 °C.

**Fig. 5:**
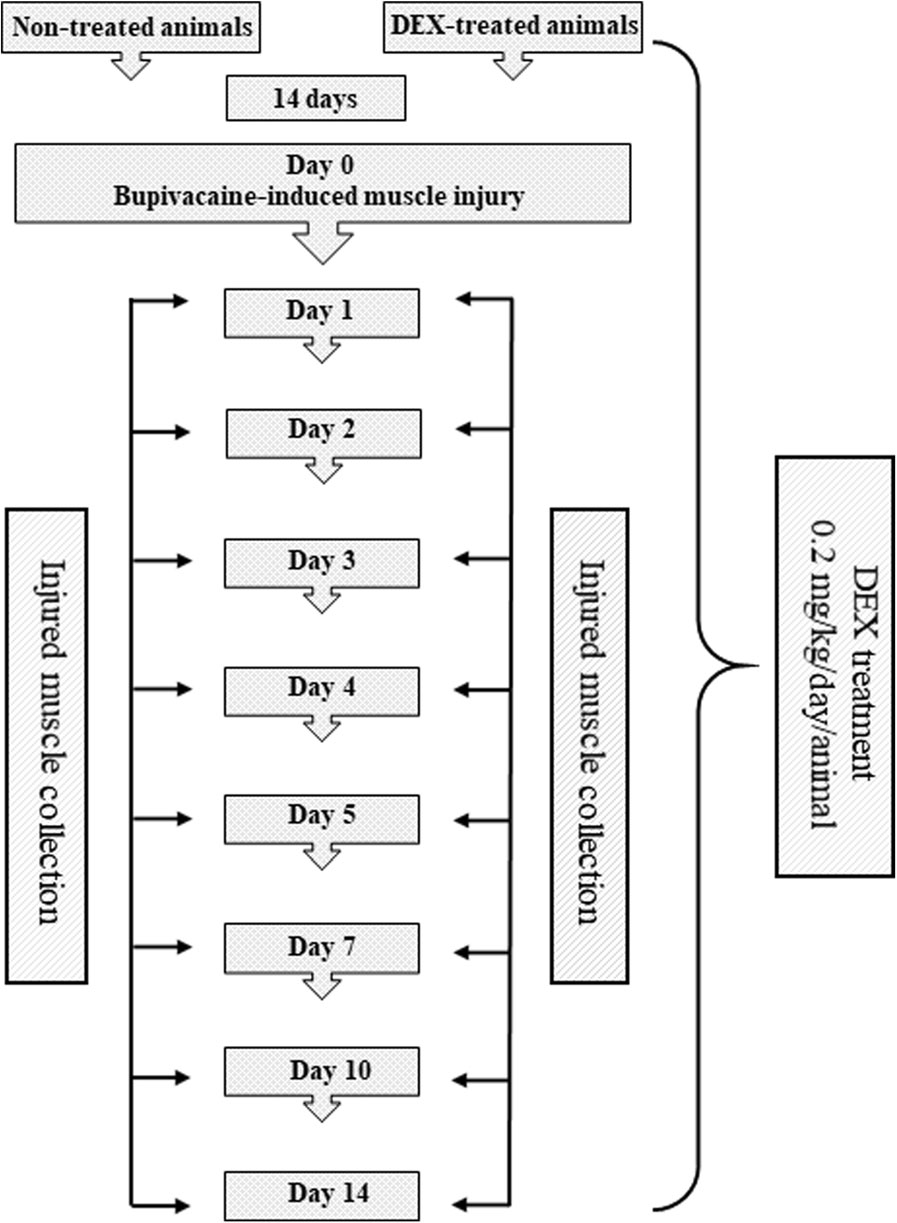
Experimental design scheme. The animals were divided into non-treated (control) and DEX-treated groups. Intramuscular DEX administration (0.2 mg/kg/day/animal) was started 14 days prior to muscle injury and was continued post-injury. On the 15th day (day 0) of the experiment, BPVC-induced muscle injury was induced. On the subsequent days after the injury, the animals were sacrificed (three gilts/group/experimental day), and muscle samples were collected for evaluation

The gilts were assigned randomly into two groups, each consisting of 24 animals: group I, non-treated (control), and group II, treated with DEX (Rapidexon 2 mg/ml, Eurovet Animal Health B.V., Bladel, The Netherlands) at a daily dose of 0.2 mg/kg from the first day to the end of the experiment. DEX was administered intramuscularly (i.m.) in the neck, perpendicular to the skin surface, just behind the base of the ear, and a hand’s width from the spine, alternately in the right and left side during following days of the experiment. The dosage of DEX was selected mainly on the basis of previous research regarding some effects of DEX using a swine model [47, 48], but also taking into account the upper dosage range of anti-inflammatory dose of DEX used in humans and the target animal species [49–51]. The animals from the control group were injected i.m. with 3.5 ml of saline solution (i.e. the average volume of DEX dose) at the corresponding site as DEX was administered. On the 15th day (day 0) of the experiment, muscle injury was induced by a 10 ml injection of 0.5% bupivacaine hydrochloride (BPVC) solution (Marcain, Astra Zeneca, UK) in both the right and left *longissimus lumborum* muscle (two independent injuries, one on the right and one on the left *longissimus lumborum* muscle per animal). The skin at the injection site was locally anaesthetized with 10% lidocaine (lidocaine spray, Egis, Budapest, Hungary) and was marked with tattoo ink. Before the muscle injury procedure (20 min), the animals were premedicated with 2 mg/kg azaperone (Stresnil, Janssen Pharmaceutica N.V., Beerse, Belgium) administered i.m. and 0.05 mg/kg atropine (Atropinum Sulfuricum, Polfa S. A, Warsaw, Poland) administered i.m. After BPVC injection, on days 1, 2, 3, 4, 5, 7, 10 and 14, the animals were euthanized (three gilts/per group/per time point) by intravenous injection (i.v.) of 0.25 ml/kg 40% pentobarbital sodium salt (Euthaminal, Alfasan, Nederland B.V). Twenty minutes before euthanasia, the animals were premedicated with 2 mg/kg azaperone (Stresnil, Janssen Pharmaceutica N.V., Beerse, Belgium) that was administered i.m. Intramuscular injections of azaperone and antropine were performed in the neck, perpendicular to the skin surface, just behind the base of the ear, and a hand’s width from the spine. The experimental study design scheme is presented in Fig. 5.

### Microscopic evaluation

The muscle samples from the injured sites of the right and left *longissimus lumborum* muscles (one site/two longitudinal and two transverse sections) were collected from each animal in both groups on days 1, 2, 3, 4, 5, 7, 10 and 14 after BPVC injection. The samples (longitudinal: approximately 4 mm thick × 10 mm wide; transverse: approximately 4 mm thick × 4 mm wide) were fixed in neutralized 10% formalin, embedded in paraffin wax and cut into 3-μm-thick sections. All longitudinal and transverse muscle sections were stained with haematoxylin (Mayer’s; Sigma-Aldrich) and eosin (Sigma-Aldrich) (H&E) for histopathological examination (evaluation of extravasation, necrosis, inflammation, MPCs, myotubes, and young myofibres).

Histologically, MPCs were identified as round or elongated cells with one, slightly oval or elongated centrally located nucleus and a scant to moderately abundant cytoplasm; they were located under the intact basal laminas at the site of myofibre injury, and prior to the fusion they began to locate one after the other. The MPCs presence was confirmed by nuclear expression of MyoD1 and cytoplasmic expression of desmin. Myotubes in H&E staining were identified as multinucleated, small in diameter cells with slightly basophilic cytoplasm, with more or less recognizable sarcomeres, their nuclei were tightly packed. Desmin expression in myotubes cytoplasm was confirmed immunohistochemically. The young myofibres were considered as multinucleated cells with initially centrally located, then peripherally located nuclei and slightly basophilic cytoplasm with distinct sarcomeric pattern confirmed by desmin immunolabelling. All antibodies used were listed in Table 1.

**Table 1:** Summary of the immunohistochemical methodology

Primary antibody	Clone	Dilution	Antigen retrieval	Visualization system
Anti-MyoD1^a^	5.8A	1:50	2 × 3 min^b^, Tris-EDTA buffer pH 9.03 min	EnVision + System-HRP, Mouse (DAB)^a^
Anti-Desmin^a^	D33	1:50	2 × 3 min^b^, Tris-EDTA buffer pH 9.03 min	EnVision + System-HRP, Mouse (DAB)^a^

^a^DAKO, Glostrup, Denmark; ^b^Antigen retrieval was conducted in a microwave oven at 650 W

Immunohistochemical labelling was performed using an immunoperoxidase method with 3,3′-diamonobenzidine (DAB) as the chromogen (Table 1). Subsequently, the sections were counterstained with haematoxylin (Mayer’s; Sigma-Aldrich). For the negative control, the primary antibody was replaced by the isotype-matched mouse IgG1 (Dako, Glostrup, Denmark) at the appropriate dilution (the negative reagent control slide was included for each test slide). For the positive control, porcine cardiac muscle (for desmin) and human rhabdomyosarcoma (for MyoD1) sections were processed together with the evaluated slides.

The course of myofibres regeneration process was evaluated on the basis of observation of the inflammatory and repair phases. In the inflammatory phase, extravasation, necrosis and inflammation were assessed, and in the repair phase, the numbers of MPCs, myotube and young myofibres were estimated. All features were assessed at 40x magnification in 10 fields of muscular injury areas in each section (two longitudinal and two transverse sections). The presence of MPCs, myotubes and young myofibres was confirmed by positive immunohistochemical labelling for the expression of MyoD1 (MPCs) and desmin (MPCs, myotubes and young myofibres), and they were assessed as the number of positive cells at 40x magnification in 10 fields in muscular injury areas in each section (two longitudinal and two transverse sections). The scoring system used to evaluate both phases of the BPVC-induced skeletal muscle regeneration was created by the authors based on their experience in research on skeletal myofibre regeneration (Table 2) [46].

**Table 2:** The scoring system used to evaluate the inflammatory and repair phases of myofibre regeneration

Category	Evaluation criterion	Points assigned per criterion
Extravasation	Absent	0
	Focal	1
	Extensive	2
Necrosis (myofibre hypercontraction, hyalinization and fragmentation of cytoplasm, loss of striations)	Absent	0
	Single fibres	1
	Focal	2
	Extensive	3
Inflammation (all inflammatory cells, i.e., neutrophils macrophages, lymphocytes, and eosinophils)	Absent	0
	Minimal: ≤ 5 inflammatory cells per HPF (40x)	1
	Mild: 6–10 inflammatory cells per HPF (40x)	2
	Moderate: 11–20 inflammatory cells per HPF (40x)	3
	Marked: > 20 inflammatory cells per HPF (40x)	4
Myogenic precursor cells (MPCs)	Absent	0
	Not numerous: ≤ 3 MPCs per HPF (40x)	1
	Moderately numerous: 4–6 MPCs per HPF (40x)	2
	Numerous: 7–12 MPCs per HPF (40x)	3
	Marked numerous: > 12 MPCs per HPF (40x)	4
Myotubes	Absent	0
	Not numerous: < 3 myotubes per HPF (40x)	1
	Moderately numerous: 3–5 myotubes per HPF (40x)	2
	Numerous: > 5 myotubes per HPF (40x)	3
Young myofibres	Absent	0
	Not numerous: < 3 young myofibres per HPF (40x)	1
	Moderately numerous: 3–5 young myofibres per HPF (40x)	2
	Numerous: > 5 young myofibres per HPF (40x)	3

### Statistical analysis

The data are expressed as the mean (±SD) score per injured muscle area. The data represent 6 sites of injured muscle per group per day [3 animals (2 independent muscle injuries per single animal per day)]. Distribution of our data was checked for normality using Shapiro-Wilk test. Student’s unpaired *t* test was used to compare the results between the DEX-treated and control groups. Statistical analysis of the kinetics (i.e., multiple comparisons between particular time points within a group) of the injury and recovery features in the DEX and control groups was performed using one-way analysis of variance (ANOVA) followed by Bonferroni’s post hoc test. Differences were determined as significant when the *p* values were < 0.05. SigmaPlot Software Version 12.0 (Systat Software Inc., San Jose, CA, USA) was used for statistical analysis and graph plotting.

## Abbreviations

BPVC: Bupivacaine hydrochloride
DAB: 3,3′-Diamonobenzidine
DEX: Dexamethasone
eNOS: endothelial nitric oxide synthase
GCs: Glucocorticoids
H&E: Haematoxylin and eosin
IGF-I: insulin-like growth factor
M1: Pro-inflammatory macrophages
M2: Anti-inflammatory macrophages
MIF: Migration inhibitory factor
MPCs: Muscle precursor cell
NO: Nitric oxide
ROS: Reactive oxygen species
TER: Transendothelial electrical resistance
